# Analysis of Dispersion of Carbon Nanotubes in *m*-Cresol

**DOI:** 10.3390/ma15113777

**Published:** 2022-05-25

**Authors:** Jaegyun Im, Dong-Myeong Lee, Jaegeun Lee

**Affiliations:** 1 School of Chemical Engineering, Pusan National University, 2 Busandaehak-ro 63 beon-gil, Geumjeong-gu, Busan 46241, Korea; spder060@gmail.com; 2 Institute of Advanced Composite Materials, Korea Institute of Science and Technology, 92 Chudong-ro, Bongdong-eup, Wanju-gun 55324, Korea; 115050@kist.re.kr; 3 Department of Organic Material Science and Engineering, Pusan National University, 2 Busandaehak-ro 63 beon-gil, Geumjeong-gu, Busan 46241, Korea

**Keywords:** carbon nanotube, *m*-cresol, dispersion

## Abstract

We analyzed the dispersion state of carbon nanotubes (CNTs) in *m*-cresol using dispersion stability analysis, optical microscopy, and UV-vis spectroscopy. The high dispersion stability of CNT/*m*-cresol dispersion was observed when it was sufficiently treated with ultrasonication. Despite the high dispersion stability, optical microscopy and UV-vis spectroscopy analysis of various CNT/*m*-cresol dispersions revealed that CNT bundles in *m*-cresol were not dispersed into individual CNTs. We also propose that the blue-shift of the G peak of CNTs in *m*-cresol in the Raman spectrum, which had been reported as evidence of the formation of the charge-transfer complex between *m*-cresol and CNTs, is rather attributed to the interference of *m*-cresol’s inherent peak at around 1600 cm^−1^.

## 1. Introduction

In the solution processing of carbon nanotubes (CNTs), their dispersion has long been a challenge. To disperse CNTs individually, various strategies, such as surfactant-assisted CNT dispersion [1,2], using the organic solvent [3], and the covalent functionalization of CNTs [4], have been tried so far, combined with physical treatments, for instance, ultrasonication [5] and the high-pressure jet homogenizer [6].

The discovery that chlorosulfonic acid (CSA) is a thermodynamic solvent for CNTs has greatly advanced the solution processing of CNTs [7]. CSA spontaneously dissolves CNTs by direct protonation and forms isotropic or various liquid crystalline phases depending on the concentration of CNTs [8]. Taking advantage of the liquid crystallinity of CNTs in CSA, highly aligned macroscopic CNT assemblies have been developed, which reported exceptional mechanical properties with electrical conductivities [9,10,11,12].

In economic processing, however, the use of CSA has several limitations. Since CSA is a super-strong acid, the processing system requires extreme safety caution. CSA also causes continuous corrosion to the equipment. Moreover, the moisture level should be strictly controlled, and the recommended dew point of the system is as low as −50 °C [8,13]. Hence, it is desirable to find a new solvent that effectively disperses CNTs under mild conditions.

Recently, *m*-cresol was reported to be an effective solvent that can disperse CNTs at a much higher concentration than other commonly used organic solvents without using additives. The mixture of CNTs and *m*-cresol can form various macroscopic phases, including pastes, gels, and doughs, as the CNT concentration increases to tens of weight percent [14]. These phases exhibit polymer-solution-like rheological and viscoelastic properties. Using the polymer-like processability, it is possible to produce various macroscopic assemblies of CNTs via appropriate processing. For example, a thin film of CNTs can be fabricated by blade coating using a CNT paste, gel phase is suitable for extrusion processing, and a dough can be transformed into arbitrary geometries by kneading or rolling. After processing, *m*-cresol can be easily removed from CNTs by washing and drying without chemical changes to either *m*-cresol or CNTs.

Using the above-mentioned processing techniques, however, it is not possible to produce macroscopic CNT assemblies of high alignment, which is essential for translating the exceptional mechanical and electrical properties of CNTs. To produce highly aligned CNT assemblies, a solution processing technique that includes shear aligning is desirable. In this processing, first, the as-supplied CNTs are disentangled, which are typically entangled at various hierarchical levels, and proper shear stresses are applied so that the CNTs are aligned [15]. For such a shear-aligning solution processing, the ability of a solvent to disperse CNTs individually is required. To test the feasibility of applying *m*-cresol to shear-aligning solution processing of CNTs, it is necessary to figure out how *m*-cresol disperses CNTs.

It is at low concentrations where we can check if a solvent disperses CNTs individually and eventually disentangles the large entangled CNT structures. Furthermore, at low concentrations, we can check if liquid crystalline phases are formed, which is beneficial for the solution processing. Here, the low concentration range indicates the typical concentration range used in the solution processing and optical microscopy analysis, which is under about 1 wt.% [1,6,16,17]. The local alignment preexisting in the liquid crystalline CNTs can be inherited to the final material, maximizing the final alignment [7]. However, the dispersion of CNTs in *m*-cresol at low concentrations has not been thoroughly analyzed.

Here, we analyze the dispersion state of CNTs in *m*-cresol at a low CNT concentration (0.5 mg/mL) via dispersion stability analysis, optical microscopy, and UV-vis spectroscopy. We found that *m*-cresol disperses CNTs stably at the low concentration but does not disperse them individually to form a homogeneous isotropic phase. We also present a new interpretation of the blue-shift of the G peak of CNTs in *m*-cresol in the Raman spectrum, with a discussion about the dispersion mechanism of CNTs in *m*-cresol.

## 2. Materials and Methods

*Materials*: Commercial single-walled and multi-walled carbon nanotube products were obtained: TUBALL (OCSiAl, Novosibirsk, Russia), SG101 (ZEON Corporation, Tokyo, Japan), and BT1001M (LG Chem, Seoul, Korea) (Table 1). *m*-cresol (99.0%) and HCl (35.0%) were purchased from Samchun Chemicals (Seoul, Korea) and Duksan Pure Chemicals (Ansan-si, Korea), respectively, and were used as received.

*Dispersion stability analysis*: For the dispersion stability analysis, two dispersion samples were prepared. Sample 1 was prepared with 20 mg of TUBALL and 40 mL of *m*-cresol for the concentration of 0.5 mg/mL and stirred at 150 rpm for 24 h with a magnetic bar. Sample 2 was prepared with the same concentration of 0.5 mg/mL and ultrasonicated using a VCX-130 sonicator (130 W, 20 kHz, Sonics and Materials, Newtown, CT, USA), equipped with a 6 mm probe at 90% amplitude for a total of 90 min without the on/off cycle mode. Each sample was mixed by shaking and 30 mL of each sample was immediately transferred to a cylindrical glass vial. Subsequently, the dispersion stability of these two samples was analyzed using Turbiscan Tower (Formulaction, Toulouse, France) for a total of 24 h, with the scanning rate of 1 scan per 2 h with a near-infrared light of 880 nm. The detection head of Turbiscan Tower scanned from the bottom to the top of a sample vial in steps of 20 μm, and the data of the transmittance and the back scattering of the near-infrared light were obtained. Since the CNT absorbs almost all of the back-scattered near-infrared light of 880 nm, the back-scattered data were not considered.

*Thermal oxidation and acid purification of carbon nanotubes*: The thermal oxidation in air was performed at 400 °C for 1 h using the quartz tube furnace. During the thermal oxidation, CNTs were placed in an alumina boat and the tube was opened throughout the process. For the acid purification, CNTs were immersed in 35% HCl at a ratio of 1 mg of CNTs per 1 mL of HCl and stirred at 80 °C for 1 h. Lastly, CNTs were neutralized with deionized water and dried overnight at 110 °C in an oven.

*Optical microscopy*, *transmission electron microscopy*, *and UV-vis spectroscopy of the CNT/m-cresol dispersion*: The CNT/*m*-cresol dispersion samples for the optical microscopy were prepared with 10 mg of the CNTs and 20 mL of *m*-cresol to make the concentration 0.5 mg/mL. The dispersion was ultrasonicated using a VCX-130 sonicator (130 W, 20 kHz, Sonics and Materials, Newtown, CT, USA) equipped with a 6 mm probe at 90% amplitude for a total of 90 min without the on/off cycle mode. Preparing the cells for the optical microscopy observation, we dropped a drop of the dispersion onto the glass slide and gently placed the cover glass on top without pressing. Then, the cells were investigated using a OSH-400PDM optical microscope (Osun Hitech, Goyang, Korea). The transmission electron microscopy (TEM) was measured using the FEI-Titan Cubed 60–300 with a Cs-Corrector and monochromator at 80 kV. The CNT/*m*-cresol dispersion sample for Figure S2a–e was prepared with 10 mg of the CNTs (TUBALL) and 20 mL of *m*-cresol to make the concentration 0.5 mg/mL, and ultrasonicated using a VCX-130 sonicator (130 W, 20 kHz, Sonics and Materials, Newtown, CT, USA) equipped with a 6 mm probe at 90% amplitude for a total of 90 min without the on/off cycle mode. The dispersion sample for Figure S2f–j was prepared with 10 mg of the CNTs (TUBALL, Leudelange, Luxembourg) and 20 mL of *m*-cresol to make the concentration 0.5 mg/mL, and ultrasonicated using the same sonicator at 20% amplitude for a total of 90 min without the on/off cycle mode. Each sample was drop-casted on the TEM grid and dried for 4 h at 110 °C in an oven. The UV-vis-nIR absorbance of CNT/*m*-cresol dispersion was measured using a V770 UV-vis-nIR spectrophotometer (Jasco, Tokyo, Japan). The CNT/*m*-cresol dispersion was prepared with 0.2 mg of TUBALL and 20 mL of *m*-cresol for the concentration of 0.01 mg/mL and ultrasonicated using a VCX-130 sonicator (130 W, 20 kHz, Sonics and Materials, Newtown, CT, USA) equipped with a 6 mm probe at 90% amplitude for a total of 60 min with 2 s/2 s on/off cycles. The dispersion was loaded to a quartz cell with the light path-length of 10 mm and sealed with Teflon stopper.

*Raman spectroscopy of the CNT/m-cresol mixture*: CNT/*m*-cresol mixtures with the concentrations of 20 and 60 mg/mL were prepared for Raman spectroscopy. The mixtures were mixed using an AR-100 planetary centrifugal mixer (THINKY, Tokyo, Japan) with the included mixing mode at 2000 rpm for 10 min. Each mixture was transferred to a glass slide and measured using a NS240-F Raman spectrometer (Nanoscope Systems, Daejeon, Korea) with a laser at excitation wavelength of 532 nm. The power of the laser was adjusted to 5% to avoid the evaporation of *m*-cresol in the mixture during the examination. *m*-cresol was measured with the same laser power of 5%. For each sample, we measured the spectrum at 10 random locations.

## 3. Results and Discussion

### 3.1. Dispersion Stability of CNTs in m-Cresol

**Figure 1:**
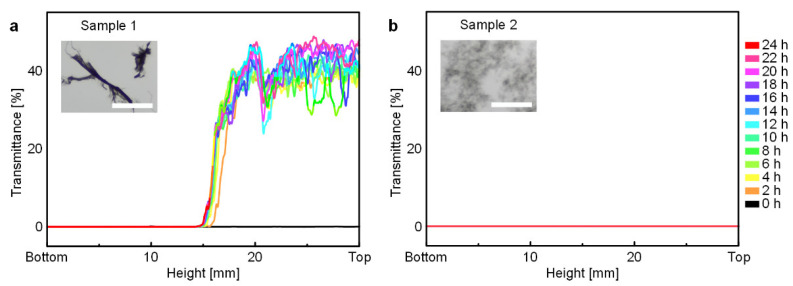
Turbiscan transmittance data of CNT/*m*-cresol dispersions. (**a**) The change of transmittance of Sample 1 (without the ultrasonication treatment) for 24 h. The optical microscopy image (inset) shows undispersed large CNT bundles. (**b**) The change of transmittance of Sample 2 (with the ultrasonication treatment) for 24 h. The optical microscopy image (inset) shows that large CNT bundles were de-bundled to small CNT bundles. Scale bars of inset optical microscopy images: 100 μm.

First, the dispersion stability of CNT/*m*-cresol dispersions treated with and without ultrasonication was analyzed by Turbiscan Tower, the dispersion stability analyzer (Figure 1). The dispersion without the ultrasonication treatment (Sample 1) had a poor dispersion stability. After 2 h from the beginning of the analysis, Sample 1 showed a gradually increasing transmittance of the dispersion at the top of the vial, whereas the transmittance of the dispersion at the bottom remained nearly zero (Figure 1a). This indicates a sedimentation of the CNTs in *m*-cresol. Furthermore, the optical microscopy images of Sample 1 show large CNT bundles (Figure 1a inset).

On the other hand, the dispersion with the ultrasonication treatment (Sample 2) showed no change of the transmittance of dispersion at all heights, even after 24 h (Figure 1b). Additionally, the optical microscopy images of Sample 2 show that large CNT bundles were de-bundled into the small CNT bundles by the ultrasonication treatment (Figure 1b inset). With these dispersion stability analysis results, we conclude that the CNT/*m*-cresol dispersion maintained a stabilized state if the dispersion was sufficiently treated with ultrasonication. Afterward, all CNT/*m*-cresol dispersions for the optical microscopy were prepared with the ultrasonication treatment.

### 3.2. Dispersion State of CNTs in m-Cresol

**Figure 2:**
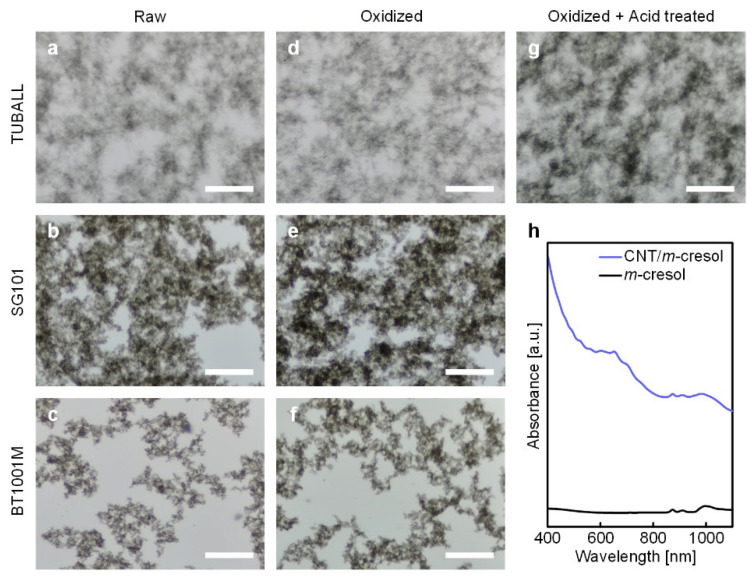
(**a**–**g**) Optical microscopy images of CNT/*m*-cresol dispersions (0.5 mg/mL): Raw (**a**) TUBALL, (**b**) SG101, and (**c**) BT1001M dispersions, oxidized (**d**) TUBALL, (**e**) SG101, and (**f**) BT1001M dispersions, oxidized and acid-treated (**g**) TUBALL dispersion. Scale bars: 100 μm. (**h**) UV-vis absorbance spectrum of CNT (TUBALL)/*m*-cresol dispersion (0.01 mg/mL).

To check whether bundles of CNTs in *m*-cresol were dispersed into individual CNTs, we analyzed the dispersion state of CNTs in *m*-cresol using optical microscopy. Since the characteristics of the CNTs may influence the dispersion state of CNT/*m*-cresol, we analyzed the dispersion state using various types of CNT products, whose characteristics are listed in Table 1. Overall, CNTs are not individually dispersed in the whole region of the dispersion, regardless of the type of CNT products (Figure 2a–c). The clouds distributed over the dispersion in the optical microscopy images in Figure 2 are the cluster of agglomerated CNTs. These agglomerated CNT clouds are usually observed when CNTs are not homogeneously dispersed, whereas the clouds are not observed when CNTs are homogenously dispersed [5]. For the comparison, the optical microscopy image of the homogeneous CNT dispersion using surfactants is shown in Figure S1. Additionally, the TEM images of the dispersion used in Figure 2a are shown in Figure S2a–e. From the TEM images, we confirmed that CNTs in the dispersion were not individually dispersed even with the harsh physical treatment (ultrasonication). Therefore, the CNT bundles distributed over the dispersion were observed as the cloudy CNT agglomerates in the optical microscopy images.

**Figure 3:**
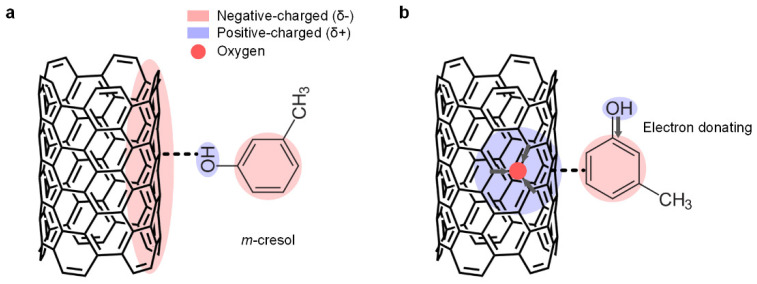
Schematic representations of the previous adsorption theories of *m*-cresol onto a CNT surface. (**a**) *m*-cresol and a CNT surface form a charge-transfer complex through *m*-cresol’s hydroxyl proton. (**b**) *m*-cresol’s negative-charged benzene ring adsorbs onto the oxygen-containing CNT basal surface.

We also tested the effect of oxidation on the dispersion behavior because the oxidation of CNTs may influence the dispersion in *m*-cresol. In fact, some theories have been proposed to explain the adsorption mechanism of *m*-cresol onto CNTs. One theory proposes that *m*-cresol and CNTs form charge-transfer complexes through *m*-cresol’s hydroxyl protons (Figure 3a) [18]. According to this theory, clean sp^2^ networks of carbon atoms are beneficial for the adsorption. On the contrary, another theory suggests that insertion of oxygen atoms in CNTs promotes the adsorption of *m*-cresol. They insist that the inserted oxygen localizes the electrons from the π-electron system of the carbon basal plane and creates the positive-charged surface around them. Consequently, the negative-charged benzene ring of *m*-cresol, induced by its electron donor-acceptor molecule structure, adsorbs onto the positive-charged carbon basal surface (Figure 3b) [19]. As we cannot rule out one scenario in favor of the other, we tested the effect of oxidation on the dispersion behavior, using CNTs with thermal oxidation or acid purification. The thermal oxidation incorporates oxygen at the surface of CNTs, as thoroughly described in the earlier work [12]. Moreover, for the acid purification after the thermal oxidation, it was reported that this purification method efficiently eliminates residual impurities [12,20,21] and amorphous carbons on the surface of CNTs [12].

Interestingly, according to the earlier work using the molecule dynamics simulation, the incorporation of oxygen moieties to the CNT surface does not increase the adsorption affinity of *m*-cresol on the CNT surface [22]. We also observed that the thermal oxidation and the acid purification of CNTs did not significantly improve the dispersibility of CNTs in *m*-cresol (Figure 2d–g), consistent with the aforementioned molecule dynamics simulation result [22]. Although *m*-cresol adsorbs on CNTs, it seems that the adsorbed *m*-cresol does not significantly screen the van der Waals force between CNTs to disperse them individually. For instance, the bile salts, a surfactant that has a semi-rigid and π-π interaction-capable cholesterol group, individually disperse CNTs better than other surfactants, securing the distance between CNTs via the flattened bean-shaped molecule feature [23]. On the contrary, due to *m*-cresol’s simple and small molecular structure, it does not secure a significant distance between CNTs to screen the strong van der Waals force of CNTs.

Additionally, we analyzed the dispersion state using UV-vis spectroscopy. The wavelength range used in Figure 2h is the part of the wavelength range that associated with the electron transitions (van Hove transitions) of CNTs. If CNTs are well-dispersed into individual CNTs in the dispersion, the electron transitions (van Hove transitions) of the CNT by the absorbate on the CNT surface occur [24]. In this case, the UV-vis spectrum of the CNT dispersion shows some absorbance peaks that indicate the existence of individually dispersed CNTs and are separated by the different diameter of CNTs. On the other hand, if the CNT dispersion is dominated by CNT bundles, the amount of electron transitions of CNTs by the absorbate will decrease. As a result, we cannot observe multiple sharp absorbance peaks. As shown in Figure 2h, the UV-vis spectrum of CNT (TUBALL)/*m*-cresol dispersion of an even lower concentration (0.01 mg/mL) did not show multiple sharp absorbance peaks, and it was similar to that of the CNT dispersion with aggregated small CNT bundles, as reported before [24].

With these results, we can conclude that *m*-cresol does not disperse CNTs individually, regardless of the oxidation state of CNTs, and that the dispersion state is governed by aggregated small CNT bundles, even with a sufficiently harsh physical dispersing treatment (ultrasonication).

### 3.3. Raman Spectroscopy of CNT/m-Cresol System

In earlier works, it was suggested that *m*-cresol and CNTs form charge-transfer complexes through *m*-cresol’s hydroxyl protons [14,18]. Generally, when electron acceptors are adsorbed on nanocarbon materials, the blue-shift of the G peak of the Raman spectrum (dG > 0) is observed [25]. From this background, as evidence of the formation of charge-transfer complex between CNTs, the blue-shift of the G peak of the Raman spectrum of CNT/*m*-cresol was provided [18]. According to this theory, we also attempted to analyze the formation of the charge-transfer complex between *m*-cresol and CNTs. However, our results were inconsistent with the previous reports.

**Figure 4:**
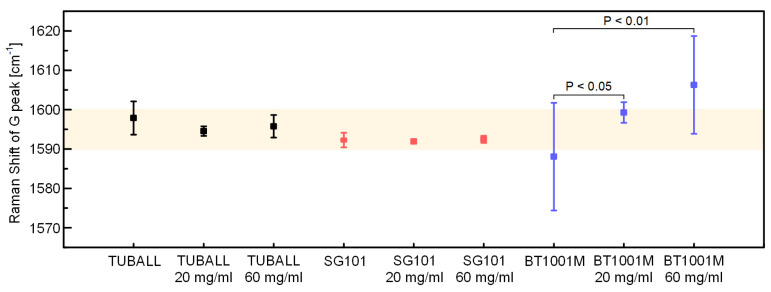
Location of G peaks of each CNT and CNT/*m*-cresol. The colored region is the range of 1590–1600 cm^−1^.

We obtained Raman spectra of various CNT samples and their mixture with *m*-cresol and measured the location of G peaks (Figure 4). The location of the G peak of the CNT/*m*-cresol mixture and that of the corresponding pure CNT samples were statistically compared by the one-sided t-test to see if there was a blue-shift in the G peak. As a result, there is no strong evidence to conclude that the G peak of the CNT/*m*-cresol mixture was blue-shifted at the level of significance of 0.05, except for the case of BT1001M, which is multi-walled (Figure 4). The exact *p*-values of CNT samples from the one-sided t-test are listed in Table S1.

**Figure 5:**
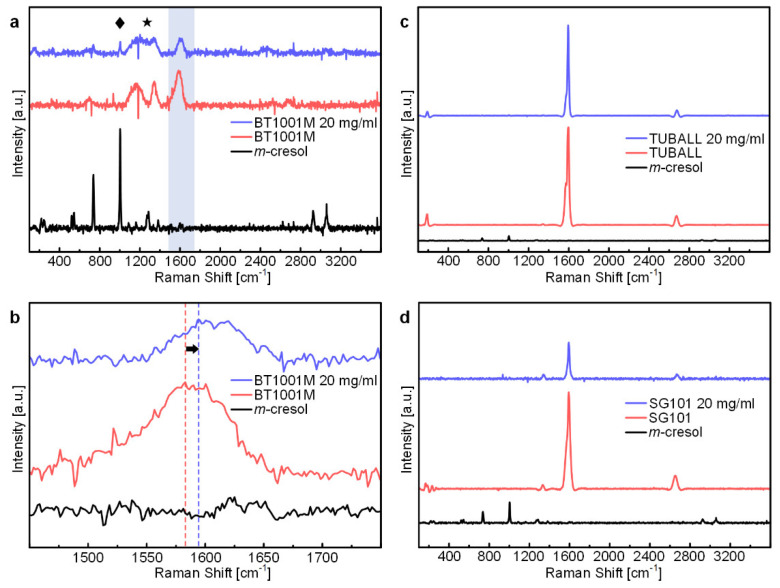
(**a**) Raman spectra of BT1001M/*m*-cresol mixture (20 mg/mL), BT1001M, and *m*-cresol. The diamond, star, and blue region indicate Raman shift ranges, those are the Raman spectrum of *m*-cresol affects that of BT1001M. (**b**) Magnified Raman spectra of the blue region (1450–1750 cm^−1^) of (**a**). (**c**) Raman spectra of TUBALL/*m*-cresol mixture (20 mg/mL), TUBALL, and *m*-cresol. (**d**) Raman spectra of SG101/*m*-cresol mixture (20 mg/mL), SG101, and *m*-cresol.

We attribute the blue-shift of the G peak of BT1001M/*m*-cresol to the Raman peak of *m*-cresol. It was reported that the Raman spectrum of pure *m*-cresol has a peak at around 1600 cm^−1^ [26]. When we analyzed the Raman spectrum of pure *m*-cresol, we also observed a peak at around 1600 cm^−1^ (Figure 5a,b). The Raman spectrum of *m*-cresol with less noises is shown in Figure S3. BT1001M had a G peak at 1588 cm^−1^, and the peak intensity was low (Figure 5a). Hence, the G peak of BT1001M may be easily affected by the peak of *m*-cresol at around 1600 cm^−1^, resulting in the blue-shift toward 1600 cm^−1^. CNT samples used in the earlier work [18] were also multi-walled and had a weak G peak in the range of 1575–1588 cm^−1^. Therefore, it seems that they could be easily influenced by *m*-cresol’s inherent peak. We also observed that the Raman spectra in other ranges were affected by the Raman spectra of *m*-cresol (see the diamond and star of Figure 5a). In contrast, the G peaks of TUBALL and SG101 had a much higher intensity (Figure 5c,d) and were located at higher Raman shifts (1597.97 and 1592.27 cm^−1^, respectively, Figure 4). For these reasons, G peaks of these products were hardly affected by the peak of *m*-cresol at around 1600 cm^−1^.

Therefore, it would be reasonable to conclude that the blue-shift of the G peak is due to the interference by *m*-cresol’s peak, especially for CNT samples, whose G peak is relatively weak. The existence of the charge-transfer complexes within the CNT/*m*-cresol system has been sufficiently confirmed with various characterization methods in earlier studies. However, as shown in our study, characterizing the charge-transfer complex only with Raman spectra of the CNT and its *m*-cresol mixture is not recommended. When analyzing the mixture of CNTs and some chemicals by the shift of the G peak, we suggest that one should consider the influence of the Raman spectra of the pure chemical and that the analysis should be performed with as many types of CNTs as possible to reach a more general conclusion.

## 4. Conclusions

The CNT/*m*-cresol dispersion with the ultrasonication treatment had excellent dispersion stability. The dispersion stability of the ultrasonicated dispersion was well-maintained after 24 h, whereas the sedimentation of CNTs in the dispersion without the ultrasonication treatment occurred right after the dispersion stability analysis began. With optical microscopy images and UV-vis spectrum of the CNT/*m*-cresol dispersion, we conclude that *m*-cresol does not disperse CNTs individually. Thus, it is difficult to apply a shear-aligning solution processing technique to CNT/*m*-cresol dispersion. Finally, we suggest that the blue-shift of the G peak of CNTs in *m*-cresol in the Raman spectrum-the proposed evidence for the charge-transfer complex-was due to the interference of *m*-cresol’s inherent peak at around 1600 cm^−1^. Careful caution is required when interpreting the shift of the G peak of CNTs in *m*-cresol.

## Figures and Tables

**Table 1:** Properties of CNT products (data were obtained from the suppliers).

CNT Product (Supplier)	TUBALL (OCSiAl)	SG101 (ZEONANO)	BT1001M (LG Chem)
CNT type	SWCNT	SWCNT	MWCNT
Specific surface area (m^2^/g)	800–1600	800	250
Length (μm)	>5	100–600	N/A
Diameter (nm)	1.6 ± 0.4	3–5	10
Carbon purity (wt.%)	99	99	95
Bulk density (kg/m^3^)	N/A	N/A	25
*I*_G_/*I*_D_ ratio *	136.0	24.7	1.5

* The *I*_G_/*I*_D_ ratio of each product was measured by the authors with the characterization method described in the experimental section of Raman spectroscopy. The highest *I*_G_/*I*_D_ ratio of each product is listed.

## Data Availability

The data presented in this study are available upon request from the corresponding author.

## Supplementary Materials

The following supporting information can be downloaded at: https://www.mdpi.com/article/10.3390/ma15113777/s1, Figure S1: The optical microscopy image of the homogenous CNT dispersion using the surfactant; Figure S2: The transmission electron microscopy images of the TUBALL/*m*-cresol dispersions; Figure S3: Raman spectrum of *m*-cresol with the laser power of 30%; Figure S4: UV-vis absorbance spectra of CNT (TUBALL)/*m*-cresol dispersions; Table S1: The results of the one-sided t-test for the difference of the location of the G peak of pure CNTs and CNT dispersion.
