# Supplementary material for: Analysis of Dispersion of Carbon Nanotubes in m-Cresol
Source: Materials (Basel). 2022 May 25;15(11):3777. doi: 10.3390/ma15113777 (PMC9181108; doi:10.3390/ma15113777)
Supplement: Supplementary file 1 [file materials-15-03777-s001.zip › materials-1723567-supplementary.pdf]

Supporting information

Analysis of dispersion of carbon nanotubes in *m*-cresol

Jaegyun Im[1], Dong-Myeong Lee[2], and Jaegeun Lee[1,3] *

[1]School of Chemical Engineering, Pusan National University, 2, Busandaehak-ro 63beon-gil, Geumjeong-gu, Busan, 46241, Republic of Korea

[2]Institute of Advanced Composite Materials, Korea Institute of Science and Technology, 92 Chudong-ro, Bongdong-eup, Wanju, Jeonbuk, 55324, Republic of Korea

[3]Department of Organic Material and Science, Pusan National University, 2, Busandaehak-ro 63beon-gil, Geumjeong-gu, Busan, 46241, Republic of Korea

*author to whom correspondence should be addressed.

E-mail address: jglee@pusan.ac.kr (Jaegeun Lee)

1. Optical microscopy image of homogenous CNT dispersion

We show the optical microscopy image of the homogenous CNT (SG101, 0.6 wt.%) dispersion using the surfactant (sodium cholate, 5.4 wt.%) and deionized water (23.5 ml) as a solvent. Compared to the CNT/*m*-cresol dispersions (Figure 2a-g), there is no clouds of agglomerated CNTs.

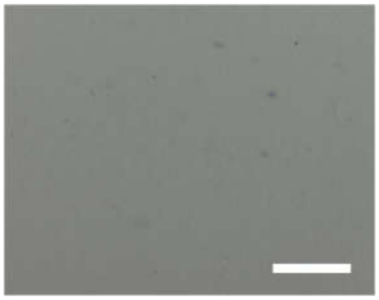

Figure S1. The optical microscopy image of the homogenous CNT dispersion using the surfactant. Scale bar: 100 μm.

2. Transmission electron microscopy of the CNT/*m*-cresol dispersions

We measured the transmission electron microscopy (TEM) of the CNT/*m*-cresol dispersions. The dispersion for Figure S2a-e is the same dispersion used in Figure 2a (TUBALL/*m*-cresol at the concentration of 0.5 mg/ml). Even with the sufficiently harsh ultrasonication treatment (90% of the sonication amplitude), the CNTs are not dispersed individually (Figure S2a). We also confirm that the CNTs are still bundled at higher magnifications (Figure S2b-e). For comparison, the dispersion at the same concentration (0.5 mg/ml) but with the low ultrasonication treatment (20% of the sonication amplitude) was also examined. We observed that it had larger bundles than the dispersion with the harsh ultrasonication treatment (Figure S2f-j).

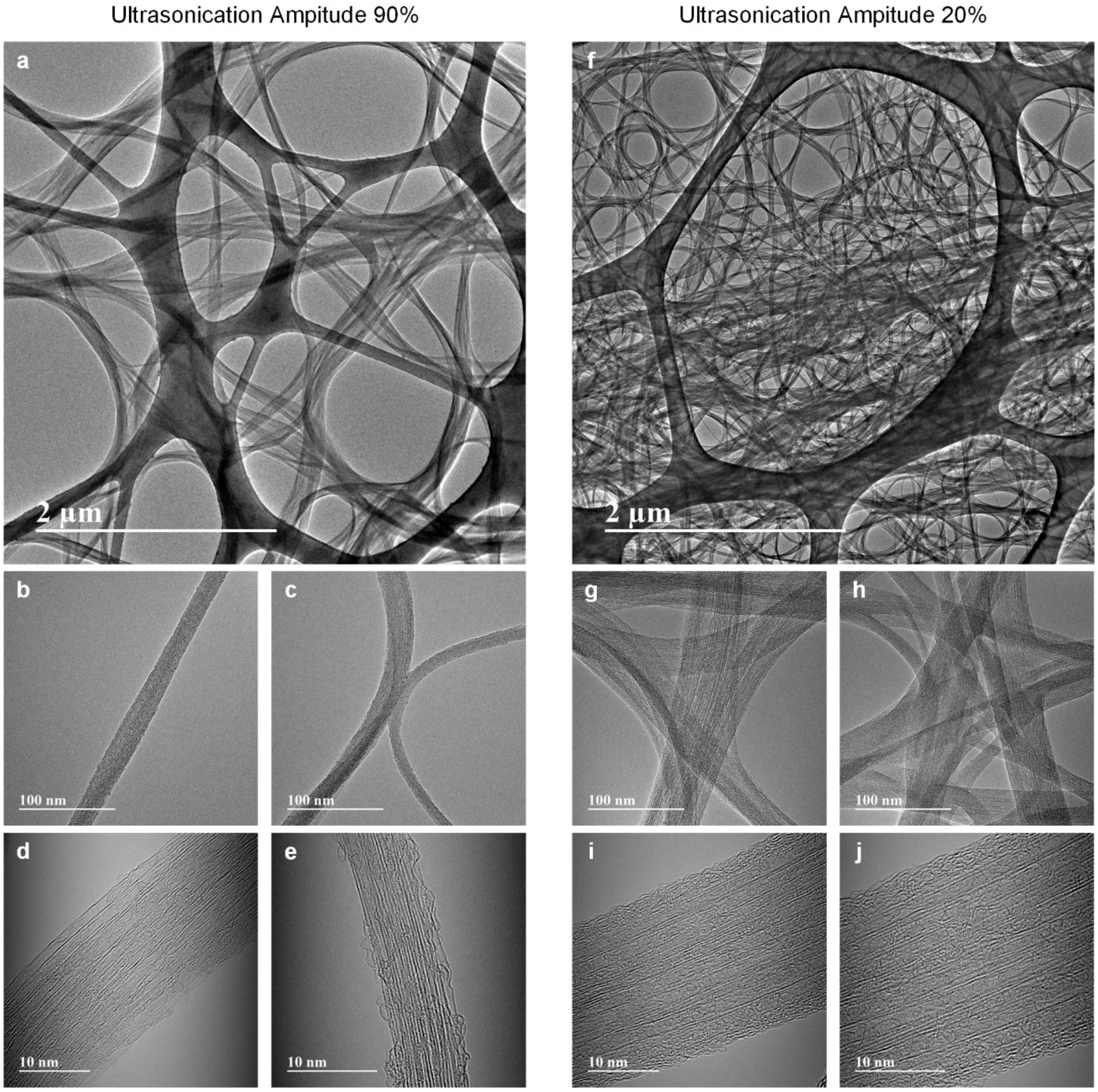

Figure S2. The transmission electron microscopy images of the TUBALL/*m*-cresol dispersions. (a-e) The dispersion with the sufficiently harsh ultrasonication treatment (90% of the sonication amplitude). (f-j) The dispersion with the low ultrasonication treatment (20% of the sonication amplitude).

3. Raman spectrum of *m*-cresol

The Raman spectrum of *m*-cresol in Figure 4 was measured with the laser power of 5%. Even though we removed the background spectrum of the dark room of the instrument, it showed low peak intensity with lots of noises. To measure the Raman spectrum of pure *m*-cresol more clearly, we measured *m*-cresol with the laser power of 30%. As a result, we obtained a clearer Raman spectrum of *m*-cresol (Figure S3). This Raman spectrum is also well consistent with the previous research [1].

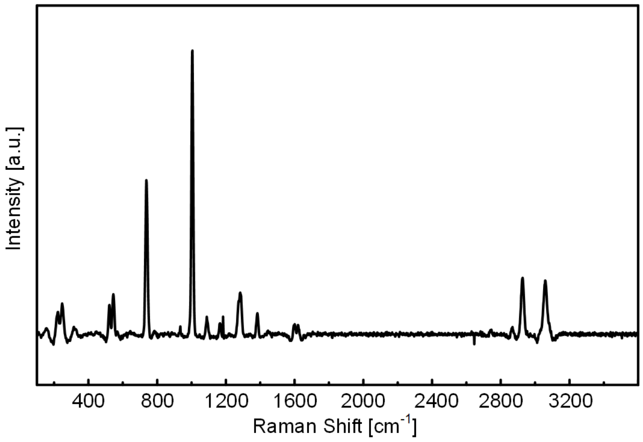

Figure S3. Raman spectrum of *m*-cresol with the laser power of 30%.

4. Statistical analysis of the shift of G peak in Raman spectrum

We define the mean of G peaks of the CNT product as μ_1 and the corresponding CNT/m-cresol mixture as μ_2. Then, we set the null hypothesis as that the difference of means between paired samples is zero ($\mu_1 - \mu_2 = 0$) and the alternative hypothesis as that the difference of means between paired samples is less than zero ($\mu_1 - \mu_2 < 0$) to confirm that the up-shift of G peaks (dG > 0) occurs in *m*-cresol. The exact P-values of CNT samples from the one-sided t-test are listed in Table S1.

Table S1. The result of the one-sided t-test for the difference of the location of G peak of pure CNTs and CNT dispersion.

Paired samples for the one-sided t-test	dG [cm^{-1}]	P-value
TUBALL & TUBALL/*m*-cresol (20 mg/ml)	-3.3263	0.9815
TUBALL & TUBALL/*m*-cresol (60 mg/ml)	-2.1009	0.8951
SG101 & SG101/*m*-cresol (20 mg/ml)	-0.3502	0.7128
SG101 & SG101/*m*-cresol (60 mg/ml)	0.1751	0.3957
BT1001M & BT1001M/*m*-cresol (20 mg/ml)	11.206	**0.0149***
BT1001M & BT1001M/*m*-cresol (60 mg/ml)	18.209	**0.0030***

* P-values less than 0.02 are marked bold.

5. UV-vis absorbance spectrum of CNT/*m*-cresol dispersions of various concentrations

We measured the UV-vis absorbance spectra of CNT (TUBALL)/*m*-cresol dispersions of various concentrations. We found that the UV-vis absorbance spectra of CNT/*m*-cresol dispersions are easily saturated at the concentrations above 0.05 mg/ml (Figure S4). As the dispersion is dominated by the CNT bundles, it can be seen that the spectrum saturates very easily even with a small increase in the concentration (0.01 mg/ml to 0.05 mg/ml). If CNTs are dispersed individually, the absorbance will gradually increase and peaks indicating van Hove singularity could be identified as we mentioned in the manuscript. We successfully measured the UV-vis absorbance spectrum only at the concentration of 0.01 mg/ml. Thus, we analyzed the dispersion state with this dispersion and described in the manuscript with the Figure 2h. All dispersion samples are prepared with the same ultrasonication treatment as described in the manuscript "2. Materials and Methods/*Optical microscopy, transmission electron microscopy, and UV-vis spectroscopy of the CNT/m-cresol dispersion*".

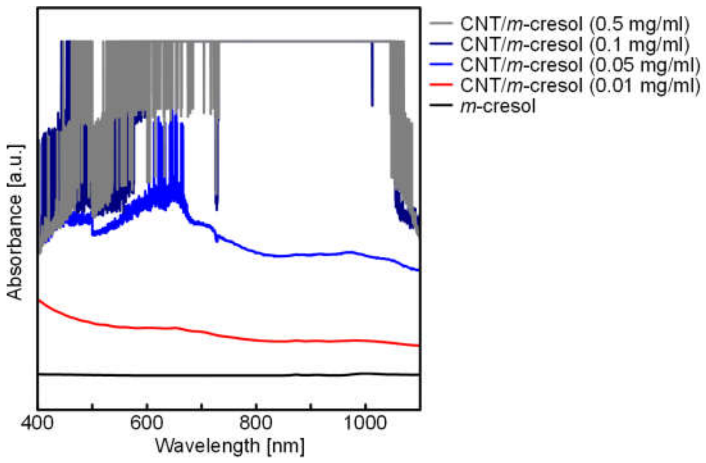

Figure S4. UV-vis absorbance spectra of CNT (TUBALL)/*m*-cresol dispersions.

Reference

1. Yu, H.; Yan, P. Determination of M-Cresol and p-Cresol in Industrial Cresols by Raman Spectrometer. In Proceedings of the Advanced Materials Research; 2012; Vol. 468–471, pp. 1104–1109.
